# High intraperitoneal interleukin-6 levels predict ultrafiltration (UF) insufficiency in peritoneal dialysis patients: A prospective cohort study

**DOI:** 10.3389/fmed.2022.836861

**Published:** 2022-08-10

**Authors:** Qianhui Song, Xiaoxiao Yang, Yuanyuan Shi, Hao Yan, Zanzhe Yu, Zhenyuan Li, Jiangzi Yuan, Zhaohui Ni, Leyi Gu, Wei Fang

**Affiliations:** ^1^Department of Nephrology, Renji Hospital, School of Medicine, Shanghai Jiao Tong University, Shanghai, China; ^2^Shanghai Center for Peritoneal Dialysis Research, Shanghai, China

**Keywords:** interleukin-6, inflammation, peritoneal dialysis, ultrafiltration capacity, ultrafiltration insufficiency

## Abstract

**Introduction:**

UF insufficiency is a major limitation in PD efficiency and sustainability. Our study object to investigate the efficacy of intraperitoneal inflammation marker, IL-6 level as a predictor of UF insufficiency in continuous ambulatory peritoneal dialysis (CAPD) patients.

**Methods:**

Stable prevalent CAPD patients were enrolled in this prospective study. IL-6 concentration in the overnight effluent was determined and expressed as the IL-6 appearance rate (IL-6 AR). Patients were divided into two groups according to the median of IL-6 AR and prospectively followed up until death, transfer to permanent HD, recovery of renal function, kidney transplantation, transfer to other centers, lost to follow-up or to the end of study (January 31, 2021). Factors associated with UF capacity as well as dialysate IL-6 AR were assessed by multivariable linear regression. Cox proportional hazards model was used to examine the association between dialysate IL-6 AR and UF insufficiency.

**Results:**

A total of 291 PD patients were enrolled, including 148 males (51%) with a mean age of 56.6 ± 14.1 years and a median PD duration of 33.4 (12.7–57.5) months. No correlation was found between dialysate IL-6 AR and UF capacity at baseline. PD duration was found positively correlated with baseline dialysate IL-6 AR, while 24h urine volume was negatively correlated with baseline dialysate IL-6 AR (*P* < 0.05). By the end of study, UF insufficiency was observed in 56 (19.2%) patients. Patients in the high IL-6 AR group showed a significantly inferior UF insufficiency-free survival when compared with their counterparts in the low IL-6 AR group (*P* = 0.001). In the multivariate Cox regression analysis, after adjusting for DM, previous peritonitis episode and 24h urine volume, higher baseline dialysate IL-6 AR (HR 3.639, 95% CI 1.776–7.456, *P* = 0.002) were associated with an increased risk of UF insufficiency. The area under the ROC curve (AUC) for baseline IL-6 AR to predict UF insufficiency was 0.663 (95% CI, 0.580–0.746; *P* < 0.001).

**Conclusion:**

Our study suggested that the dialysate IL-6 AR could be a potential predictor of UF insufficiency in patients undergoing PD.

## Introduction

Peritoneal dialysis (PD) is a well-established and highly cost-effective treatment modality for patients with end-stage kidney disease (ESKD). Compared to hemodialysis (HD), PD achieves similar outcomes but the drop-out rate remains high (1). UF insufficiency (previously named UF failure) is a main cause for PD discontinuation, and is also associated with poor outcome of PD patients (2, 3). UF insufficiency can be present shortly after the onset of PD. Approximately 4% of incident PD patients developed early UF insufficiency (<2 years), has been suggested previously (4). Studies of peritoneal structure and function indicate that early UF insufficiency is always associated with peritoneal small-solute transport rate (PSTR) related to increased density of the peritoneal microvasculature. The prevalence of late UF insufficiency (>2 years) has been reported to be 21 and 36% in long-term PD patients (4, 5). Increasing evidence show that acquired UF insufficiency is not only associated with fast PSTR, but also related to decreased osmotic conductance to glucose (OCG) due to scarring of the vessels and interstitium (6, 7). The pathologic mechanism underlying such alterations remain unknown, but appears to be related to chronic inflammation induced by continuous exposure to bioincompatible PD fluids (PDF) and uremia, possibly exacerbated by episodes of peritonitis (8–10).

Interleukin-6 (IL-6) is a key player in modulating inflammation. IL-6 is a chief stimulator of the production of most acute phase proteins in response to various stimuli, and also plays an important role in regulating the transition from acute to chronic inflammation (11, 12). It is well-established that local high production of IL-6 is related to a persistent low-grade inflammation in the peritoneal cavity and its level increased with therapy duration (13, 14). There is increasing evidence that dialysate IL-6 level is closely associated with baseline PSTR (15–17), and it has been shown that IL-6 polymorphisms were related to inherent high PTSR in patients undergoing PD (18, 19). Moreover, we have previously reported that high intraperitoneal IL-6 levels at baseline were a predictor of increasing PSTR after 12 months follow-up in PD patients (20). The above results suggest that IL-6 not only correlates with PSTR at the start of PD, but also can affect the alteration of PSTR during prolonged PD treatment. However, whether intraperitoneal IL-6 level can predict the UF capacity still remain unclear.

Therefore, we conducted the present study to investigate the association between baseline dialysate IL-6 AR and UF insufficiency in patients undergoing PD.

## Materials and methods

### Study population

Stable CAPD patients in Renji Hospital, School of Medicine, Shanghai Jiao Tong University, between January 2014 and April 2015 were recruited in present study. Exclusion criteria included: (1) presence of systemic inflammatory disease including chronic autoimmune disorders, peritonitis or acute infections that requires antibiotic therapy in the preceding 3 months; (2) malignancy; (3) taking glucocorticoid or immunosuppressive agents during the past 1 year; (4) acute cardiocerebrovascular events that occurred within 3 months prior to the study; (5) patients with PD catheter malfunction and/or fluid leaks; (6) patients with UF insufficiency; (7) patients who refused to give consent. All enrolled patients were dialyzed using glucose-based PD solutions (Dianeal^®^, Baxter). The protocol of study was approved by the Ethics Committee of Renji Hospital, School of Medicine, Shanghai Jiao Tong University, China (number: [2013] N022; year: January/2014). All the participants signed informed consent before enrollment.

### Data collection and patient evaluation

The demographic characteristics collected at the enrollment of study included: age, gender, height, weight, date of PD initiation, underlying cause of ESKD, presence of comorbid diseases such as diabetes mellitus (DM) and cardiovascular disease (CVD), taking angiotensin-converting enzyme inhibitor/ angiotensin receptor blocker (ACEI/ARB) or not. CVD was defined as a previous history of coronary artery disease, peripheral vascular disease or cerebrovascular disease. The body surface area (BSA) was calculated by the Du Bois equation (21). The historical dialysis regimen was collected to calculate the amount of historical glucose exposure according to Davies et al. (22). At study enrollment, we also measured serum albumin, high sensitivity CRP (hs-CRP) and hemoglobin levels of each patient, and blood pressure was measured twice at an interval of 5 min to take the average.

At enrollment, all patients were asked to perform a standard peritoneal equilibration test (PET) as originally described by Twardowski (23) and PD effluent (PDE) samples were collected at baseline. The detailed procedure for undertaking a standard PET is shown in Supplementary material 1. On the night prior to PD center visit, patients did a dialysis exchange using his or her usual overnight dialysis regimen. The overnight effluent was fully drained the next morning in the PD center. A 10-mL sample was collected from the drained PDF and immediately stored at −80°C. We weighed the bag of drained effluent to assess the volume and recorded the dwell duration. The 24h ultrafiltration and 24h urine output of each patient were measured. UF capacity was estimated based on the net negative balance (weighing the bag after drainage) after a 2 L 2.5% glucose exchange with 4 h of dwell time in the PET. Residual renal function (RRF) was calculated as an average of 24h urine urea and creatinine clearance (24, 25). Small solute clearance was assessed by 24-h dialysate and urine collection, with the calculation of total weekly Kt/V and weekly CrCl normalized to 1.73 m^2^ body surface area (26). Mass transfer area coefficient for creatinine (MTACcr) was calculated using the simplified Garred equation (27). A validated correction factor was used to calculate peritoneal protein clearance (Prcl) (28). The IL-6 concentration in the drained PDF were measured at baseline, and patients were divided into 2 groups according to the median of IL-6 AR. All patients re-performed PET at 12 monthly intervals and UF capacity (4h net ultrafiltration, mL) was recorded from baseline to 72 months.

### Determination of IL-6 in PD effluent

The concentration of IL-6 in the drained PDF was determined with enzyme-linked immunosorbent assay (ELISA). All samples were run simultaneously and in duplicate to avoid intra- and inter- assay variations. The IL-6 concentration was measured by Human IL-6 ELISA Ready-SET-Go! (eBioscience^®^, CA, USA). Due to the concentrations of dialysate cytokines were influenced by UF capacity which was affected by peritoneal solute transfer rate and dwell time, the dialysate appearance rate (AR) was calculated as dialysate concentration times the drained volume divided by the dwell time and expressed as pg (ng) per minute [pg (ng)/min].

### Study outcomes

All patients were prospectively followed up until death, transfer to permanent HD, recovery of renal function, kidney transplantation, transfer to other centers, lost to follow-up or to the end of study (January 31, 2021). The primary outcome measures in our study were UF insufficiency, and the second outcome measures in our study were technique failure. During the study period, all UF insufficiencies and technique failures from patient enrollment to study endpoint were carefully recorded. Other PD outcomes including death, transplant and transfer to other centers were also collected. In our study, UF insufficiency was defined according to the International Society for Peritoneal Dialysis guidelines: that is, net UF from a 4-h PET is <100 mL (2.27% glucose /2.5% dextrose) (29). PD catheter malfunction and/or fluid leaks were ruled out prior to the diagnosis of UF insufficiency, and when signs of catheter malfunction and/or leaks occurred, the cause of catheter dysfunction was determined by some combination of physical examination, abdominal radiography and peritoneography, as required. In patients who developed catheter dysfunction, conservative therapy was given initially: supine position and a lower infusion volume for leaks; abdominal massage, administration of aperients or enemas, or ambulation for malposition; clot dislodgement with heparin or urokinase for obstruction; and administration of aperients or enemas for omental wrap. Technique failure was defined as dialysis modality switch from PD to HD for at least 3 months.

### Statistics analysis

Kolmogorov–Smirnov test was applied to test normal distributions. Data were described by mean ± SD, median and inter quartile range (IQR), or proportions as appropriate. Unpaired *t*-tests or Mann-Whitney tests were used to compare different groups depending on whether the data were normally distributed or skewed. Categorical variables were compared using chi-square tests. Multiple linear regression analysis was performed to assess the predictors for UF capacity as well as dialysate IL-6 AR. Kaplan-Meier analysis was used to compare UF insufficiency-free survival time between the low IL-6 AR group and the high IL-6 AR group. Risk factors associated with UF insufficiency were determined by univariate and then by a multivariate Cox proportional hazards model. Only covariates that remained significant (*P* < 0.05) in the univariate analysis were kept in the multivariate Cox regression model except those with collinearity. Collinearity was assessed using the variance inflation factors method. The area under the curve (AUC) of dialysate IL-6 AR for incident UF insufficiency was calculated by receiver operating characteristic (ROC) analysis. Given the large number of statistical tests performed, *P*-values were adjusted using the Benjamini-Hochberg (BH) procedure for false discovery rate control in multivariate regression analysis (30, 31). Data analysis was carried out using the SPSS software package (version 22.0, Chicago, IL, USA) and R software (version 3.6.1, Vienna, Austria; the p.adjust function was used to obtain adjusted *P*-value). All probabilities were two-tailed and a *p* < 0.05 indicated significance.

## Results

### Patient demography and membrane function

**Figure 1:**
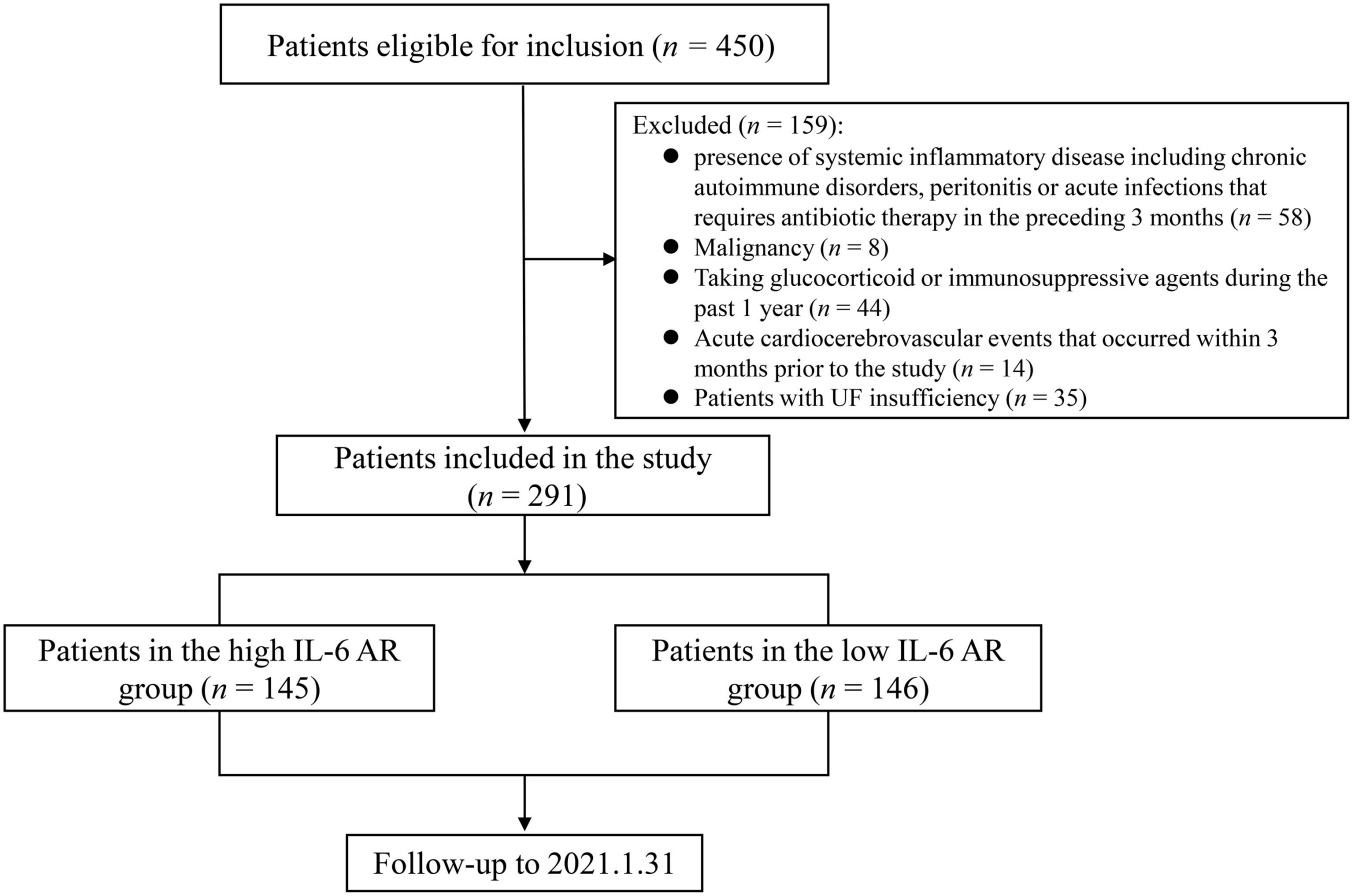
Flowchart of patient inclusion in analysis. AR, appearance rate.

The flowchart of patients in the study was shown in Figure 1. Detailed characteristics of the study population at enrollment were summarized in Table 1. Based on inclusion and exclusion criteria, a total of 291 patients were enrolled, representing 65% of the total PD population in our center, including 148 males (51%) with a mean age 56.6 ± 14.1 years and a median PD duration 33.4 (12.7–57.5) months. Among the 291 patients, 73 (25%) patients had diabetes mellitus as comorbidity. UF capacity at enrollment for the entire study cohort was 306.8 ± 124 mL. In comparison to patients with low IL-6 AR, those with high IL-6 AR levels above the median were more likely to have experienced a peritonitis episode in the past (*P* < 0.05) and had longer PD duration [45.3 (20.4–76.2) vs. 20.3 (5.1–45.5) months, *P* < 0.001], higher historical glucose exposure [47,862 (40,150, 57,128) vs. 40,150 (32,850, 51,332) g/year, *P* < 0.001] as well as higher 24h ultrafiltration [650 (298–938) vs. 423 (-25–696) mL, *P* < 0.001] whereas low IL-6 AR patients had higher urine output [500 (48–1,000) vs. 80 (0–500) mL, *P* < 0.001] and higher RRF [1.34 (0–3.49) vs. 0.73 (0–2.39) mL/min, *P* = 0.032]. Other demographic characteristics, laboratory data and peritoneal membrane function were similar between the low IL-6 AR group and high IL-6 AR group (see Table 1).

**Table 1:** Characteristics of the study population (*n* = 291).

**Variable**	**All PD patients**	**Low IL-6 AR group**	**High IL-6 AR group**	***P*-value**
	**(*n* = 291)**	**(*n* = 146)**	**(*n* = 145)**	
Age (years)	56.4 ± 14.1	56.3 ± 14.8	56.5 ± 13.4	0.922
Gender (Male)	148 (51%)	74 (51%)	74 (51%)	0.952
BSA (m^2^)	1.62 ± 0.17	1.62 ± 0.17	1.62 ± 0.16	0.935
Systolic pressure (mmHg)	140 ± 21	140 ± 21	139 ± 22	0.548
Diastolic pressure (mmHg)	87 ± 13	87 ± 12	87 ± 13	0.864
PD duration (months)	33.4 (12.7–57.5)	20.3 (5.1–45.5)	45.3 (20.4–76.2)	<0.001
Underlying renal disease [n (%)]				
Chronic glomerulonephritis	86 (30%)	49 (34%)	37 (26%)	0.133
Diabetic nephropathy	37 (13%)	16 (11%)	21 (15%)	0.367
Hypertension	12 (4%)	4 (3%)	8 (6%)	0.233
Polycystic kidney disease	8 (3%)	4 (3%)	4 (3%)	1.000
Obstructive nephropathy	4 (1%)	1 (1%)	3 (2%)	0.610
Others and Unknown	147 (51%)	72 (49%)	75 (52%)	0.681
Comorbidity [*n* (%)]				
Diabetes mellitus	73 (25%)	35 (24%)	38 (26%)	0.660
Hypertension	276 (95%)	138 (95%)	138 (95%)	0.801
Cardiovascular disease	112 (39%)	61 (42%)	51 (35%)	0.247
ACEI/ARB taking [*n* (%)]	155 (53%)	78 (53%)	77 (53%)	0.956
Previous RRT [*n* (%)]	2 (0.7%)	1 (0.7%)	1 (0.7%)	1.000
Previous peritonitis episode [*n* (%)]	71 (24%)	24 (16%)	47 (32%)	0.002
Historical glucose exposure (g/year)	43,800 (33,150, 55,293)	40,150 (32,850, 51,332)	47,862 (40,150, 57,128)	<0.001
Hemoglobin (g/L)	107.4 ± 16.8	107.3 ± 16.1	107.6 ± 17.5	0.888
Serum albumin (g/L)	37.2 ± 4.4	37.0 ± 4.6	37.3 ± 4.2	0.625
Hs-CRP (mg/L)	2.38 (0.8–6.56)	1.96 (0.76–5.6)	2.89 (0.87–6.91)	0.251
Dialysis adequacy				
Total Kt/V urea	1.94 ± 0.36	2.01 ± 0.39	1.87 ± 0.31	0.104
Peritoneal Kt/V urea	1.58 ± 0.36	1.54 ± 0.37	1.62 ± 0.35	0.138
Renal Kt/V urea	0.18 (0–0.61)	0.29 (0–0.87)	0.16 (0–0.40)	0.076
Total CrCl (L/week/1.73 m^2^)	61.2 ± 18	65.7 ± 20.6	57.4 ± 13.9	0.117
RRF (mL/min)	0.92 (0–2.89)	1.34 (0–3.49)	0.73 (0–2.39)	0.032
Urine output, mL/24 h	300 (0–800)	500 (48–1000)	80 (0–500)	<0.001
UF, mL/24 h	510 (100–840)	423 (-25–696)	650 (298–938)	<0.001
nPCR (g/Kg/day)	0.88 ± 0.18	0.91 ± 0.18	0.85 ± 0.18	0.205
4h D/Pcr	0.61 ± 0.11	0.61 ± 0.11	0.61 ± 0.12	0.819
4h UF (mL)	306.8 ± 124	318.9 ± 128.2	294.5 ± 118.8	0.094
MTACcr (mL/min)	7.46 (6.02–9.29)	7.54 (6.14–9.31)	7.36 (5.74–9.43)	0.788
Prcl (mL/d)	68.6 (52.3–90.4)	68.2 (52.3–88.3)	68.8 (52.6–92.3)	0.571

*Values expressed as mean ± standard deviation, median (25–75th percentile), or absolute numbers with percentages [n (%)].*

*BSA, body surface area; ACEI/ARB, inhibitor/ angiotensin receptor blocker; RRT, renal replacement therapy; Hs-CRP, high-sensitivity high sensitivity C-reactive protein; Kt/Vurea, urea kinetics; CrCl, creatinine clearance; RRF, residual renal function; nPCR, normalized protein catabolic rate; D/Pcr, peritoneal transport characteristics; UF, ultrafiltration; MTACcr, mass transfer area coefficient for creatinine; Prcl, peritoneal protein clearance*.

### Factors associated with UF capacity

**Figure 2:**
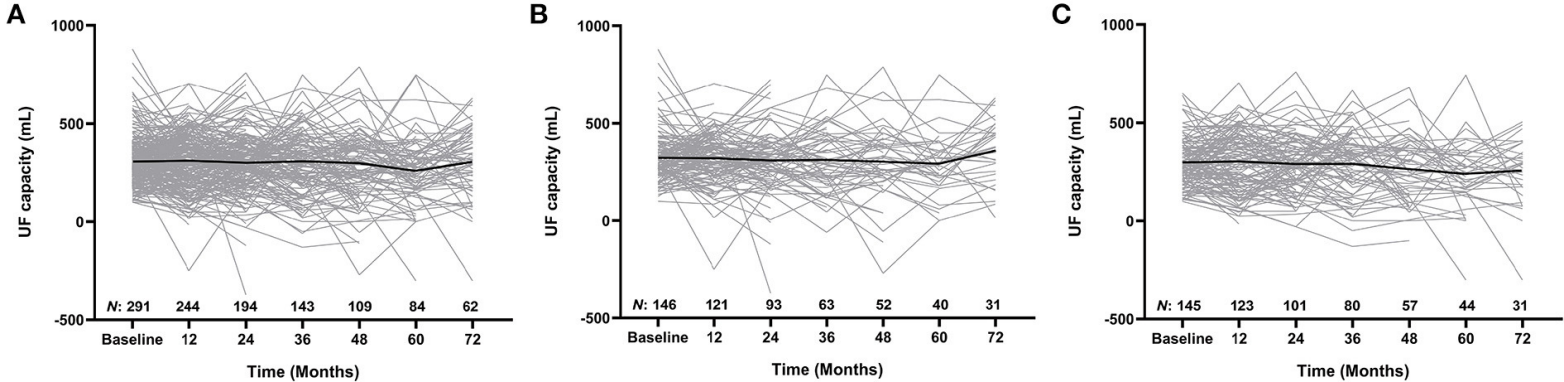
Longitudinal changes in UF capacity for the whole cohort **(A)**, low IL-6 AR group **(B)**, and high IL-6 AR group **(C)**. Values expressed as trajectories of UF capacity for each single patient with an overlay of mean curves. UF, ultrafiltration; AR, appearance rate.

UF capacity (mL) as well as the numbers of patients at each time point (i.e., every 12 months over a total of 72 months) on PD were presented in Figure 2, and the comparison of peritoneal membrane function between patients in the low IL-6 AR group and high IL-6 AR group was shown in Supplementary Table 1. Multivariable linear regression showed that after adjustment for age, gender, PD duration, serum albumin, hs-CRP, historical glucose exposure and previous peritonitis episode, comorbid with diabetes (β = −0.128, *P* = 0.038), 24h urine volume (β = −0.192, *P* = 0.006) and dialysate IL-6 AR (β = −0.144, *P* = 0.022) correlate (inversely) with UF capacity at enrollment (see Table 2). However, this association lost statistical significance after being corrected for multiple testing.

**Table 2:** Association of log_10_ dialysate IL-6 AR and 24h urine volume with UF capacity at baseline.

	**Unit increase**	**Standardized β coefficient**	***t***	***P*-value**	***P*-value^a^**
**Model constant**			3.698	0.000	
Age	1 year	0.020	0.316	0.752	0.823
Gender	-	0.030	0.499	0.618	0.772
PD duration	1 year	−0.085	−1.163	0.246	0.492
DM	-	−0.128	−2.088	0.038	0.127
Historical glucose exposure	1,000 g/year	−0.035	−0.604	0.546	0.772
Previous peritonitis episode	-	0.015	0.224	0.823	0.823
Plasma albumin	1 g/L	0.078	1.261	0.208	0.492
Log_10_ hs-CRP	10 mg/L	0.062	1.034	0.302	0.503
24h urine volume	100 ml	−0.192	−2.768	0.006	0.060
Log_10_ dialysate IL-6 AR	10 pg/min	−0.144	−2.295	0.022	0.110

a *BH-adjusted P-value.*

*BSA, body surface area; DM, diabetes; Hs-CRP, high-sensitivity high sensitivity C-reactive protein; AR, appearance rate; BH, Benjamini-Hochberg*.

### Factors associated with dialysate IL-6 AR level

The effluent level of IL-6 AR in this cohort was 55.1 (35.1–102.7) pg/min and the CVinter was 132%. Multivariable linear regression showed that after adjustment for age, gender, DM, serum albumin, hs-CRP, previous peritonitis episode and historical glucose exposure, PD duration (β = 0.176, *P* = 0.049) had a positive correlation with baseline dialysate IL-6 AR, while 24h urine volume (β = −0.235, *P* < 0.001) had a negative correlation with baseline dialysate IL-6 AR after being corrected for multiple testing (see Table 3).

**Table 3:** Association of 24h urine volume with log_10_ dialysate IL-6 AR at baseline.

	**Unit increase**	**Standardized β coefficient**	***T***	***P*-value**	***P*-value^a^**
**Model constant**			8.313	0.000	
Age	1 year	0.014	0.226	0.822	0.822
Gender	-	−0.087	−1.539	0.125	0.375
PD duration	1 year	0.176	2.552	0.011	0.049
DM	-	0.041	0.712	0.477	0.821
Historical glucose exposure	1,000 g/year	0.074	1.339	0.182	0.410
Previous peritonitis episode	-	0.029	0.449	0.654	0.822
Plasma albumin	1 g/L	−0.035	−0.603	0.547	0.821
Log_10_ hs-CRP	10 mg/L	0.013	0.236	0.814	0.822
24h urine volume	100 ml	−0.235	−3.641	<0.001	<0.001

a *BH-adjusted P-value.*

*BSA, body surface area; DM, diabetes; Hs-CRP, high-sensitivity high sensitivity C-reactive protein; BH, Benjamini-Hochberg*.

### Dialysate IL-6 AR level and outcome events

**Figure 3:**
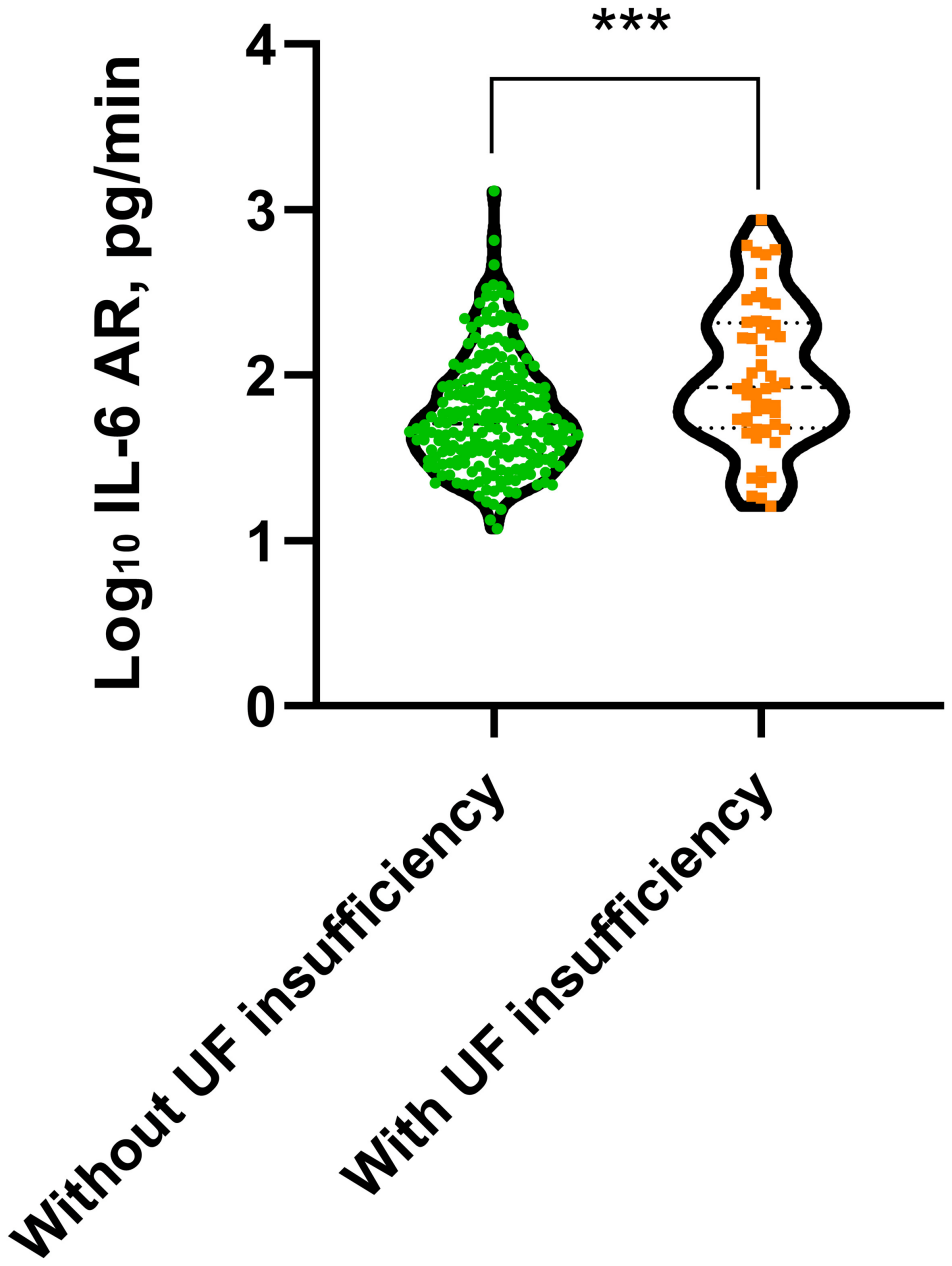
Dialysate IL-6 AR level in patients with or without UF insufficiency (***p < 0.001). Non-parametric data were compared using Mann–Whitney test. UF, ultrafiltration; AR, appearance rate.

Patient outcomes were summarized in Table 4. The median follow-up duration was 52.5 (IQR 24.5–79.2) months for the low IL-6 AR group and 47.5 (IQR 25.2–76.1) months for the high IL-6 AR group, respectively. During the study period, UF insufficiency was documented in 56 (19.2%) patients (low IL-6 AR *n* = 18; high IL-6 AR *n* = 38). The time to diagnosis UF insufficiency was 43.3 (IQR 22.3–77) months which corresponding to PD duration of 77.4 (IQR 42.2–101.9) months in the low IL-6 AR group, while in the high IL-6 AR group, the time to diagnosis UF insufficiency was 36.4 (IQR 17.7–70.9) months which corresponding to PD duration of 88.4 (IQR 56.8–128.6) months. UF insufficiency was more likely to be observed in patients in the high IL-6 AR group when compared with patients in the low IL-6AR group (*P* = 0.003, Table 4). In addition, baseline dialysate IL-6 AR levels were significantly higher in patients who experienced UF insufficiency at any time during the study period when compared to their counterparts who remained their UF capacity (*P* < 0.001, Figure 3). By the end of study, 62 (21.3%) patients (low IL-6 AR *n* = 27; high IL-6 AR *n* = 35) experienced technique failure. The leading cause of technique failure was peritonitis (48.4%), followed by UF insufficiency (27.4%), personal preferences (12.9%), encapsulating peritoneal sclerosis (4.8%), retroperitoneal leak (3.2%), and unknown reasons (3.2%). High IL-6 AR patients experienced more technique failures due to UF insufficiency compared to low IL-6 AR patients (*P* = 0.001, Table 4), and there was no significant difference of other causes in these two groups.

**Table 4:** Follow-up and outcome events in the patients by IL-6 AR.

**Variable**	**All PD patients**	**Low IL-6 AR group**	**High IL-6 AR group**	***P*-value**
	**(*n* = 291)**	**(*n* = 146)**	**(*n* = 145)**	
**Follow-up (months)**	50.8 (24.9, 78.4)	52.5 (24.5, 79.2)	47.5 (25.2, 76.1)	0.545
**UF insufficiencies**, ***n*** **(%)**	56 (19.2)	18 (12.3)	38 (26.2)	0.003
**Technique failure**, ***n*** **(%)**	62 (21.3)	27 (18.5)	35 (24.1)	0.240
Peritonitis	30 (48.4)	15 (55.6)	15 (42.9)	1.000
UF insufficiency	17 (27.4)	2 (7.4)	15 (42.9)	0.001
Personal preferences	8 (12.9)	6 (22.2)	2 (5.7)	0.282
EPS	3 (4.8)	0	3 (8.6)	0.122
Retroperitoneal leak	2 (3.2)	2 (7.4)	0	0.498
Unknown	2 (3.2)	2 (7.4)	0	0.498
**Other outcome**, ***n*** **(%)**				
Death	102 (35.1)	50 (34.2)	52 (35.9)	0.773
Still on PD	85 (29.2)	48 (32.9)	37 (25.5)	0.167
Transplant	32 (11.0)	18 (12.3)	14 (9.7)	0.517
Transfer to other centers	9 (3.1)	3 (2.1)	6 (4.1)	0.335
Lost to follow-up	1 (0.3)	0	1 (0.7)	1.000

*Non-parametric data were compared using Mann–Whitney test and categorical variables were compared using chi-square tests.*

*AR, appearance rate; PD, peritoneal dialysis; EPS, encapsulating peritoneal sclerosis*.

### Association between dialysate IL-6 AR level and UF insufficiency

**Figure 4:**
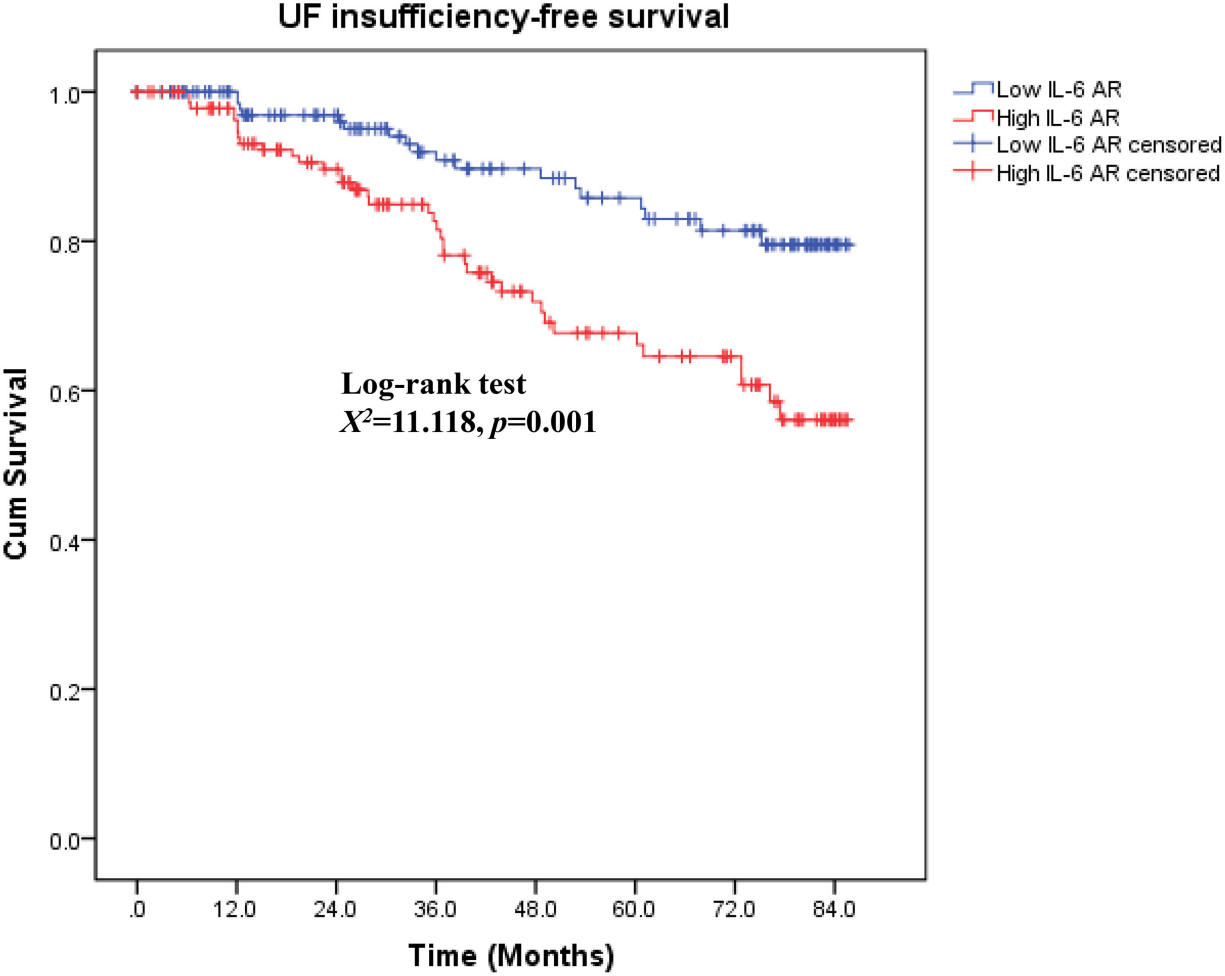
Kaplan–Meier curves by IL-6 AR for UF insufficiency-free survival. UF, ultrafiltration; AR, appearance rate.

**Figure 5:**
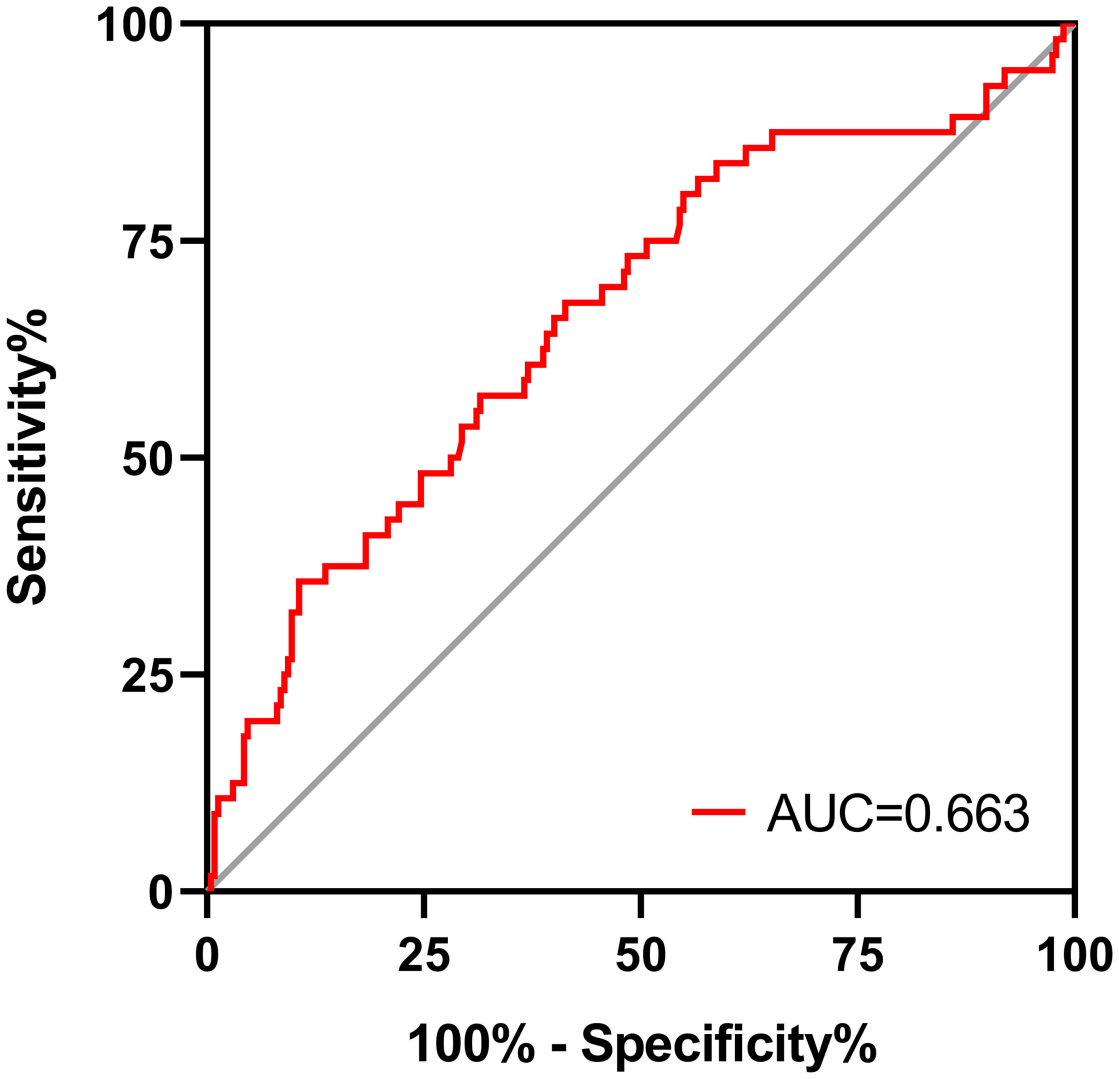
ROC curve for dialysate IL-6 AR to predict UF insufficiency in PD patients. AR, appearance rate; UF, ultrafiltration.

As shown in Figure 4, patients in low IL-6 AR group had better UF insufficiency-free survival than that in patients with high IL-6 AR (Log-rank *X*^2^ = 11.118, *P* = 0.001). UF insufficiency-free survival was 98, 91, and 84% at 1, 3, and 5 years in the low IL-6 AR group and 95, 82, and 66% at 1, 3, and 5 years in the high IL-6 AR group, respectively. Multivariate Cox proportional hazards modeling showed that after adjusting for DM, previous peritonitis episode and 24h urine volume, higher baseline dialysate IL-6 AR levels (HR 3.639, 95% CI 1.776–7.456, *P* = 0.002) were associated with an increased risk of UF insufficiency after being corrected for multiple testing (see Table 5). As shown in Figure 5, the area under the ROC curve (AUC) of the model used for baseline IL-6 AR to predict UF insufficiency was 0.663 (95% CI, 0.580–0.746; *P* < 0.001). The optimal cut-off value to discriminate UF insufficiency was 59.6 pg/mL for dialysate IL-6 AR (67.9% sensitivity, 58.7% specificity, *P* < 0.001).

**Table 5:** Predictors of UF insufficiency on Cox regression analysis.

	**Univariate**		**Multivariate**		
	**HR (95% CI)**	***P*-value**	**HR (95% CI)**	***P*-value**	***P*-value^a^**
Age (per year)	1.018 (0.996, 1.040)	0.109			
Gender (male)	0.820 (0.482, 1.395)	0.464			
BSA (per 1 m^2^)	2.104 (0.414, 10.678)	0.370			
PD duration (per year)	1.004 (0.999, 1.010)	0.138			
DM (yes)	1.808 (1.006, 3.250)	0.048	1.711 (0.948, 3.086)	0.074	0.148
CVD (yes)	0.991 (0.574, 1.912)	0.975			
ACEI/ARB taking (yes)	1.415 (0.829, 2.415)	0.204			
Previous peritonitis episode (yes)	1.876 (1.081, 3.255)	0.025	1.409 (0.772, 2.571)	0.263	0.351
Historical glucose exposure (per 1000 g/year)	1.000 (0.997, 1.002)	0.702			
Serum albumin (per 10 g/L)	0.970 (0.912, 1.033)	0.345			
Hemoglobin (per 10 g/L)	0.992 (0.976, 1.008)	0.318			
Log_10_ hs-CRP (per 10 mg/L)	1.261 (0.807, 1.971)	0.308			
24h urine volume (per 100 mL)	0.999 (0.999, 1.000)	0.024	1.000 (0.999, 1.000)	0.620	0.620
Log_10_ dialysate IL-6 AR (per 10 pg/min)	4.503 (2.329, 8.707)	<0.001	3.639 (1.776, 7.456)	<0.001	0.002

a *BH-adjusted P-value.*

*HR, hazard ratio; CI, confidence interval; BSA, body surface area; DM, diabetes; CVD, cardiovascular disease; ACEI/ARB, inhibitor/angiotensin receptor blocker; Hs-CRP, high-sensitivity high sensitivity C-reactive protein; AR, appearance rate; BH, Benjamini-Hochberg*.

## Discussion

To the best of our knowledge, this is the first study suggesting the efficacy of a single measurement of intraperitoneal IL-6 level in predicting development of UF insufficiency in patients undergoing PD.

UF insufficiency is a common and important, but poorly-explained complication of PD, especially in long-term patients. It has been reported that UF insufficiency accounts for ~30% of all cases of technique failure (9). The impaired UF points to the development of peritoneal membrane changes during long-term PD treatment. The typical anatomic alterations included submesothelial fibrosis, hyalinizing vasculopathy, and neoangiogenesis (6). Functionally, the peritoneal changes are reflected by an increase in small solute transfer and a reduction in the osmotic conductance of the membrane (8, 9). Current insights indicate that the changes in peritoneal structure and function involve a number of intertwined pathophysiologic mechanisms. Chronic inflammation is the most likely culprit (32–34). IL-6 is a critical biomarker of ongoing intraperitoneal inflammation in PD patients (35, 36). It has been reported that intraperitoneal IL-6 is a strong predictor of increasing PSTR in PD patients (16, 20). However, whether IL-6 level in dialysate was related to UF capacity decline needs further study.

Our study showed that dialysate IL-6 AR had a skewed distribution with wide range (11.8–1,295 pg/min) in our cohort and the CVinter was 132% in effluent IL-6-AR. In line with our study, Barreto et al. (37) reported similar results. In their study, the dialysate IL-6 AR levels of patients with short (≤ 24 months) and long (≥25 months) PD duration were 15.5–220.0 and 6.9–956.4 pg/min, respectively, with a CVinter 141%. We found that patients with high IL-6 AR were more likely to be anuric, more prevalent in previous episodes of peritonitis, and with a longer PD duration, higher historical glucose exposure and more ultrafiltration when compared with their counterparts in the low IL-6 AR group. Many factors have been claimed as contributors to peritoneal inflammation during PD, including the bioincompatibility of conventional PD solutions and peritonitis (15, 38). It has been shown that conventional PDF could induce IL-6 synthesis by peritoneal membrane cells (39, 40). An increase in intraperitoneal IL-6 concentrations with longer PD duration (i.e., at 24 months) has also been well-documented by the balANZ trial (median 7.22 vs. 31.35 pg/mL, *P* < 0.001) (41) and the extension study of the Balnet trial (conventional 47 ± 31.2 vs. 121 ± 69 pg/mL, *P* < 0.001; biocompatible 57.6 ± 54.5 vs. 143 ± 69.6 pg/mL, *P* < 0.001) (42). Several studies have found that intraperitoneal IL-6 levels are increased before and during peritonitis and remain high level even several months after clinical cure of peritonitis (35, 43). A retrospective observational study included 31 PD patients had reported that patients who developed peritonitis had higher baseline dialysate IL-6 level (58.4 ± 12.6 vs. 20.3 ± 8.7 pg/mL, *p* = 0.07) than that in patients who remained peritonitis-free (15). Our findings also indicated that the bioincompatible factors of PDF and peritonitis produce pro-inflammatory milieu in the intra-peritoneal cavity. Deterioration in UF capacity over time have been reported in PD patients and have been mainly attributed to the bio-incompatible nature of conventional PDF (44–46). The onset of a decline in UF capacity has been reported to occur at 2–4 years after PD commencement (5). In fact, several recent studies have demonstrated that peritoneal UF in PD patients may remain stable with time on treatment in relation to use of biocompatible PDFs with neutral pH and low GDP concentration as well as preserved RRF (47–49). Given the changes and the importance of urine volume and membrane transport characteristics, taking these parameters in account might better display the longitudinal change of UF capacity. However, these data were not collected in present study.

In the present study, no significant correlation was found for dialysate IL-6 AR with UF capacity at baseline. In addition, we found that PD duration positively correlated with baseline dialysate IL-6 AR, while 24h urine volume inversely correlated with baseline dialysate IL-6 AR. A number of factors potentially contribute to local peritoneal inflammation. It has been shown that bioincompatible factors in PDF could induce IL-6 synthesis and secretion by peritoneal membrane cells (39, 50). In the NEPP study, patients with a regimen low in glucose and GDPs had significantly lower IL-6 levels in overnight effluents when compared to that in patients with conventional lactate-buffered PDF (sPD regimen) (51). These studies indicated that bioincompatible factors in PDF could directly or indirectly promote local peritoneal inflammation. For patients with less urine output, higher dialysis doses or higher glucose concentrations is required to achieve sufficient solute and fluid removal. Besides, numerous studies have shown that a reduction in RRF may aggravate the chronic inflammatory state due to decreased renal clearance of various inflammatory cytokines (52, 53). Therefore, the negative effects of urine volume on intraperitoneal IL-6 might be explained by more exposure to bio-incompatible PD solutions and less efficient removal of inflammatory cytokines. The use of new, more biocompatible PD solutions and preservation of RRF might be helpful to ameliorate intraperitoneal inflammation.

During the study period, UF insufficiency was observed in 56 (19.2%) patients. UF insufficiency was more likely to be observed in high IL-6 AR group than those in low IL-6 AR group. In accordance with other studies (2, 4), peritonitis and UF insufficiency are the two main reasons for technique failure in our PD cohort. Furthermore, patients with high IL-6 AR were more likely to occur technique failure due to UF insufficiency when compared to their counterparts in low IL-6 AR group. The dialysate IL-6 AR were significantly higher in patients who underwent UF insufficiency when compared to those who remained their UF capacity. Consistent with our findings, Xiao et al. (54) also reported that the levels of IL-6 (either plasma or dialysate) in patients with UF insufficiency were significantly higher than those in patients without UF insufficiency. Our study showed that dialysate IL-6 AR could identify UF insufficiency with optimal cut-off values of 59.6 pg/mL. However, the AUC for IL-6 AR was rather weak, suggesting that it was probably not the ideal biomarker as a prognostic indicator of UF insufficiency. The underlying causes might be inter-individual variation of IL-6, lack of repeated measurements and increased peritoneal concentrations could be due to mesothelial cell injury by repeated exposures to bioincompatible PDF but also secondary to peritonitis episodes. Moreover, whether mesothelial cell injury was a prerequisite for UF insufficiency need further validation. Peritoneal biopsy studies also showed that chronic exposure of the peritoneum to conventional PDF was associated with loss of mesothelial cell monolayer (55, 56), which may result in persistently low dialysate IL-6 level in some patients with long-term PD due to reduced IL-6 synthesis and secretion. These factors may account for the poor discriminative potential for IL-6 AR to identify UF insufficiency. Also, we found that low IL-6 AR patients showed better UF insufficiency-free survival than high IL-6 AR patients. Furthermore, high baseline dialysate IL-6 AR was the only risk factor associated with UF insufficiency, while PD duration and historical glucose loads was not associated with UF insufficiency in this population. These results points to the differences that occur between patients in the course of inflammatory reaction triggered by chronic exposure to bioincompatible PDF and subsequent adverse peritoneal remodeling. There are few studies trying to find out the risk factors of UF insufficiency in PD patients. Selgas et al. (57) reported that diabetic state and higher glucose requirement to obtain adequate UF might be responsible for UF insufficiency. However, their study did not measure inflammation markers, such as CRP or IL-6. Taken together, although there was no significant correlation between IL-6 AR and UF capacity at baseline, we found that the intraperitoneal inflammation marker, IL-6 AR level rather than time on PD could predict subsequent development of UF insufficiency.

UF capacity in PD patients has been considered to be dependent on two major peritoneal components: the microcirculation and the interstitial tissue. Biopsy specimens taken from PD patients have confirmed that an increase in peritoneal vascular density in conjunction with submesothelial and perivascular fibrosis were observed in patients with membrane failure (6–8). It has long been recognized that the formation of new vessels will increase the vascular surface area, leading to rapid dissipation of the osmotic gradient and lower UF (9). Our prior study also showed that a decline in UF capacity could be partially abrogated by antiangiogenic therapy in a rat model (58). There is strong evidence that peritoneal angiogenesis is closely linked with inflammation through local IL-6. Yang et al. (50) showed that IL-6 trans-signaling could upregulate the protein expression and secretion of vascular endothelial growth factor (VEGF) in mesothelial cells. Catar et al. (59) also reported that IL-6 links inflammation with angiogenesis through the trans-signaling pathway to upregulate mesothelial VEGF production in the peritoneal membrane. In addition, the deposition of interstitial fibers will increase resistance to fluid flux, resulting in a decrease in water flow through the interstitium (6). Recent findings support the notion that chronic inflammation plays an important role in the initiation and progression of interstitial fibrosis in the peritoneal membrane (9, 10). Animal models of PD suggested that IL-6 signaling in recurrent peritoneal inflammation was key driving tissue fibrosis (60). In our previous study, we reported that blockade of IL-6 trans-signaling could attenuate peritoneal fibrosis by inhibiting the activation of Smad2/3, with reduced expressions of α-smooth muscle actin (α-SMA) and collagen type I (Col I) in a mouse model (61). Furthermore, Lambie et al. (62) showed that dialysate IL-6 levels were independently associated with the occurrence of encapsulating peritoneal sclerosis (EPS). Therefore, IL-6 may serve as a new non-invasive biomarker and a potential therapeutic target of membrane failure, and may guide clinical decisions such as timely transfer to HD in PD patients.

However, the conclusions that can be drawn from the present study are challenged by several limitations. First, this was a single center study, therefore, our results might not be generalized to other populations. Second, the cytokine levels were measured only once at enrollment. The study design did not account for potential variations of IL-6, thus a single IL-6 measurement as a predictor of outcome should be interpreted cautiously. Also, a single time point of effluent IL-6 may not reflect changes over time. The presence of longitudinal surveillance of effluent IL-6 level in PD patients and repeated assessments may provide more solid information. Thirdly, although the reasons and distributions of the informative censoring between the two groups were same, the inclusion of prevalent PD patients and high censorship rates might introduce bias in our study. Also, in the multivariate regression model, despite adjustment for historical glucose exposure at baseline, the differing observation periods might introduce bias in our study and the association between IL-6 AR and the risk of UF insufficiency might be exaggerated because the HRs would be overestimated. In addition, there might be residual confounding because of the unfeasibility to address differing observation periods in the analysis. Therefore, a well-designed, adequately powered multicenter randomized controlled clinical trial (RCT) is required to confirm the association between dialysate IL-6 AR and UF insufficiency.

In conclusion, the results from the present study suggest the intraperitoneal inflammation marker, dialysate IL-6 AR level could be a predictor of UF insufficiency in patients undergoing PD.

## Data availability statement

The original contributions presented in the study are included in the article/Supplementary material, further inquiries can be directed to the corresponding author.

## Ethics statement

The studies involving human participants were reviewed and approved by Ethics Committee of Renji Hospital, School of Medicine, Shanghai Jiao Tong University, China (number: [2013] N022; year: January/2014). The patients/participants provided their written informed consent to participate in this study. Written informed consent was obtained from the individual(s) for the publication of any potentially identifiable images or data included in this article.

## Author contributions

QS participated in the design of the study, analysis of data, and draft the manuscript. XY, YS, HY, and ZL participated in clinical data collection. ZY and JY helped to perform the statistical analysis. ZN and LG guided and supported this study. WF conceived of the study and participated in its design and coordination and helped to draft the manuscript. All authors contributed to the article and approved the submitted version.

## Funding

This work was supported by the National Basic Research Program of China (Grant Nos. 81370864 and 81670691) and Shanghai Municipal Education Commission-Gaofeng Clinical Medicine (Grant No. 20152211).

## Conflict of interest

The authors declare that the research was conducted in the absence of any commercial or financial relationships that could be construed as a potential conflict of interest.

## Publisher's note

All claims expressed in this article are solely those of the authors and do not necessarily represent those of their affiliated organizations, or those of the publisher, the editors and the reviewers. Any product that may be evaluated in this article, or claim that may be made by its manufacturer, is not guaranteed or endorsed by the publisher.
