# Supplementary material for: High intraperitoneal interleukin-6 levels predict ultrafiltration (UF) insufficiency in peritoneal dialysis patients: A prospective cohort study
Source: Front Med (Lausanne). 2022 Aug 10;9:836861. doi: 10.3389/fmed.2022.836861 (PMC9400905; doi:10.3389/fmed.2022.836861)
Supplement: Supplementary file 1 [file Data_Sheet_1.pdf]

Detailed procedure for undertaking a standard PET:

(1) PDF was routinely retained for 8-12 h the night before PET;

(2) The patient was asked to sit, and the PDF retained the night before was drained out (within 20 min). The total amount of drainage was weighed and the dwell time was recorded;

(3) 2L 2.5% glucose concentration PDF with was prepared and warmed to 37℃;

(4) The patient was instructed to take supine position, 2L 2.5% PDF was poured into the peritoneal cavity every 400ml (every 2min) and the patient was instructed to change position. and when the PDF was completely poured, the time was recorded as 0h;

(5) Collect dialysate samples at 0h: 200 ml of dialysate was drained from the peritoneal cavity, shaken 2~3 times, disinfected the dosing port, 10 ml dialysate was extracted with a syringe, and the remaining 190 ml was poured back into the peritoneal cavity;

(6) Retained for 2h and dialysate samples were collected the same as 0h, blood samples were collected for 2h at the same time;

(7) Retained for 4h and collected dialysate samples: the patient was instructed to sit and drain all the PDF (within 20 min), weigh the total amount of drainage, shake the dialysis bag 2-3 times, and extract 10 ml dialysate with a syringe;

(8) The concentrations of creatinine and glucose in the dialysate and blood collected above were determined.
