# Supplementary material for: High intraperitoneal interleukin-6 levels predict ultrafiltration (UF) insufficiency in peritoneal dialysis patients: A prospective cohort study
Source: Front Med (Lausanne). 2022 Aug 10;9:836861. doi: 10.3389/fmed.2022.836861 (PMC9400905; doi:10.3389/fmed.2022.836861)
Supplement: Supplementary file 2 [file Table_1.pdf]

Supplementary Table 1. Comparison of peritoneal membrane function between patients in the low IL-6 AR group and high IL-6 AR group

**Variable**	**All patients (n = 291)**	**Low IL-6 AR group (n = 146)**	**High IL-6 AR group (n = 145)**	**P value**
UF capacity (baseline) mL	306.75±124.0	218.90±128.2	294.52±118.8	0.094
UF capacity (12 months) mL	308.0±142.3	317.6±130.8	298.5±152.8	0.300
UF capacity (24 months) mL	307.8±132.1	305.8±158.7	286.0±145.8	0.367
UF capacity (36 months) mL	293.6±155.9	308.6±158.1	288.6±160.4	0.466
D/P cr at 4 h (baseline)	0.61±0.11	0.61±0.11	0.61±0.12	0.819
D/P cr at 4 h (12 months)	0.61±0.11	0.60±0.11	0.62±0.12	0.160
D/P cr at 4 h (24 months)	0.61±0.11	0.59±0.11	0.63±0.13	0.033
D/P cr at 4 h (36 months)	0.66±0.13	0.64±0.12	0.67±0.12	0.077
MTACcr (baseline) mL/min	7.46 (6.02,9.29)	7.53 (6.14, 9.31)	7.36 (5.74, 9.43)	0.791
MTACcr (12 months) mL/min	7.54(5.88-9.88)	7.14 (5.49, 9.20)	7.72 (6.00, 10.36)	0.143
MTACcr (24 months) mL/min	7.29 (6.00, 9.89)	6.93 (5.69, 9.35)	7.49 (6.27, 11.05)	0.026
MTACcr (36 months) mL/min	8.55 (6.74, 11.50)	8.50 (6.53, 10.81)	8.74 (6.92, 12.43)	0.164
Prcl (baseline) mL/d	68.6 (52.9, 90.4)	68.2 (52.6, 88.3)	68.8 (52.6, 92.3)	0.572
Prcl (12 months) mL/d	73.9 (58.9, 102.8)	73.2 (58.5, 104.4)	76.3(60.2, 101.9)	0.564
Prcl (24 months) mL/d	82.6 (63.3, 104.0)	73.6 (54.9, 102.0)	89.6 (66.7, 105.7)	0.054
Prcl (36 months) mL/d	66.5 (55.9, 84.8)	62.4 (52.8, 77.8)	72.3 (61.2, 88.0)	0.004

Abbreviations: UF: ultrafiltration; D/Pcr: peritoneal transport characteristics; MTACcr: mass transfer area coefficient for creatinine; Prcl: peritoneal protein clearance.
